# Sexual dimorphism of the posterior condylar offset of the femur and the medial posterior slope of the tibia in non-arthritic knees of Egyptian adults: an MRI study

**DOI:** 10.1186/s13018-023-03833-2

**Published:** 2023-05-12

**Authors:** Mohammad Kamal Abdelnasser, Mohammed Anter Abdelhameed, Micheal Bassem, Mahmoud Faisal Adam, Hatem M. Bakr, Yaser E. Khalifa

**Affiliations:** Orthopedic and Traumatology Department, Assiut University Hospital, Assiut, Egypt

**Keywords:** MRI, Posterior condylar offset of the femur, Posterior condylar offset of the femur ratio, Posterior slope of the tibia, Sexual dimorphism, Total knee arthroplasty

## Abstract

**Background:**

The aim of this magnetic resonance imaging (MRI) study was to investigate controversial sexual dimorphism of the posterior condylar offset of the femur (the offset) and the posterior slope of the tibia (the slope) in non-arthritic knees of Egyptian adults.

**Methods:**

On 100 male and 100 female MRIs of non-arthritic knees, linear measurements of the distal part of the femur (the offset) and the angular measurements of the proximal part of the tibia (the slope) were performed and compared regarding sex and ethnicity. The intraclass correlation coefficient (ICC) was used to test the interrater agreement.

**Results:**

Both offsets and the lateral offset ratio were larger in males (*p* < 0.001), the medial offset ratio, and the medial slope in females (*p* from < 0.001 to 0.007), whereas the lateral slope was sex-free (*p* = 0.41). Irrespective of sex, however, the medial offset with its ratio, and the medial slope were larger than their counterparts (*p* < 0.001). Our means of the offsets, their ratios, and the slopes mostly differed from those of other ethnicities (*p* from ≤ 0.001 to 0.004). ICCs > 0.8 proved MRI’s precision was high.

**Conclusion:**

There was a sexual dimorphism of both the offset and the medial slope in non-arthritic knees of Egyptian adults. We believe future designs of knee implants should consider these differences in order to improve postoperative range of motion and patients’ satisfaction after total knee arthroplasty.

*Level of evidence* Level III Retrospective Cohort Study.

*Trial registration* ClinicalTrials.gov identifier: NCT03622034, registered on July 28, 2018.

## Background

Bellemans et al. [1] were the first to define the posterior condylar offset of the femur (»the offset«) as the "maximal thickness of the posterior condyle, projected posteriorly to the tangent of the posterior cortex of the femoral shaft" as measured on true lateral radiographs of the knee. Restoration of the offset after total knee arthroplasty (TKA) is important to re-establish normal knee mechanics, maximize range of motion (ROM), and prevent impingement aiming for reduction in knee flexion instability [2, 3].

Several studies exhibited variations in the anthropometric measurements of the knee among different ethnicities [4–10]. Many have reported a sex difference in knee anthropometrics, forming the basis of the sex specific knee [5, 11–14]. Ethnic and sex differences were correspondingly noted in the population of the Middle East regarding both arthritic [15] and non-arthritic knees [16]. In a similar fashion, ethnic and sex differences of the offset and the posterior slope of the tibia (»the slope«) were demonstrated by few [9, 17–19], whereas others perceived no sex divergence [4].

Some researchers stressed the importance of restoring the offset [1–3, 20, 21] and the slope [21–25] in order to reinstate knee flexion after TKA. On the contrary, others found no relation of the offset [26–31] and the slope [31] with regard to knee flexion, though anatomical studies found them to be correlated [7, 32].

Hence, given their contribution to knee flexion after TKA and the fact Middle and Far Eastern populations need longer periods of knee flexion owing to religious and social conventions [33], special attention must be paid to restore the normal or nearly normal values of the offset and the slope after TKA.

Our clinical hypothesis was the offset and the slope differed between sexes. Therefore, the aim of this magnetic resonance imaging (MRI) study was to investigate controversial sexual dimorphism of the posterior condylar offset of the femur and the posterior slope of the tibia in non-arthritic knees of Egyptian adults.

## Patients and methods

### Patients

This was a single-center observational cross-sectional imaging study. Following ethical approval, MRIs of adult patients with suspected knee ligamentous injury in the period between 2017 and 2019 were eligible for inclusion. However, knees with imaging signs of osteoarthritis (albeit grade I), bony or cartilaginous defects, ligamentous injuries as well as those of patients with body mass index (BMI) ≥ 25 were excluded. Of the remaining total, 200 knees were classified as healthy, matched according to age into equal sex groups (100 males, and 100 females) and included in the imaging study.

### Methods

Two senior orthopedic surgeons (MAA and MB) performed the measurements. To determine the interrater agreement, the measurements were repeated on 60 randomly chosen images two weeks after the initial ratings.

The imaging technique was published earlier [16]. All MRI measurements were performed on T_2_-weighted images in the sagittal plane [34].

#### The posterior condylar offset and the posterior condylar offset ratio of the femur

**Fig. 1:**
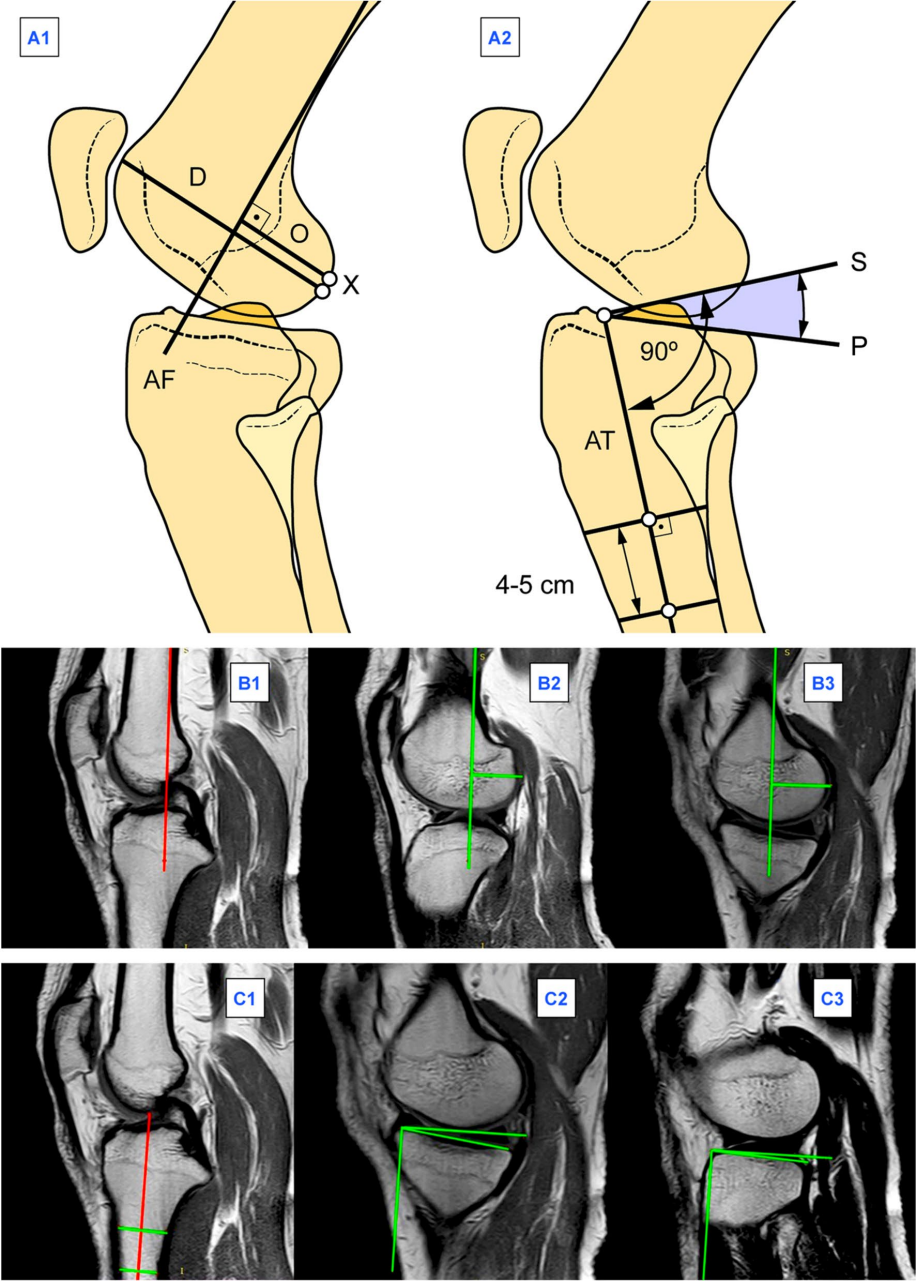
Schematic (**A**) and MRI (**B** and **C**) measurement of the posterior condylar offset and the posterior condylar offset ratio of the femur (**A1**, **B1**–**B3**) and the posterior slope of the tibia (**A2**, **C1**–**C3**) on each side of the right knee. *AF* posterior cortical axis of the femur, *AT* anatomical axis of the proximal part of the tibia, *D* longest anteroposterior diameter of the femoral condyle, *R* ratio of the longest anteroposterior diameter of the femoral condyle and the posterior condylar offset of the femur, *O* posterior condylar offset of the femur, *P* tangent of the tibial plateau, *S* posterior slope of the tibia (slope’s tangent), *X* most posterior femoral point of the outer margin of the condylar cartilage

The reference image was the middle image (Fig. 1B1). On the reference image, we drew the posterior cortical axis of the femur and reproduced it onto the image showing the most posterior projection of the respective femoral condyle on each side. For each femoral condyle, the offset was a vertical distance (in millimeters) connecting the posterior cortical axis of the femur and the most posterior femoral point of the outer margin of the condylar cartilage [34]. For each femoral condyle, the offset ratio was calculated as the fraction of the offset and the longest anteroposterior diameter of the femoral condyle [35] (Fig. 1A1; B1–B3).

#### The posterior slope of the tibia

The reference image was the image showing the insertion of the posterior cruciate ligament (Fig. 1C1). On the reference image, we drew the anatomical axis of the proximal part of the tibia (the line connecting the midpoints of two horizontals 4–5 cm apart) and reproduced its parallel onto the mid-condylar image to which the slope’s tangent was vertical on each side. For each tibial condyle, the slope was an angle (in degrees) between the tangent of the tibial plateau, and either the slope’s tangent to the most of the superior bone profile on the lateral or the slope’s tangent to the upper portion of the anterior and posterior bone profile on the medial side [36] (Fig. 1A2; C1–C3).

#### Statistical analysis

We used G Power (v. 3.1.9.7.; Franz Faul, Kiel University, Germany) to calculate statistical power a priori, and SPSS™ (v. 12.0.; IBM Corporation, Somers, NY USA) to test the difference between averaged measurements using parametric (one sample mean test, and independent samples t-test), and nonparametric (Mann–Whitney-U test) statistical tests.

To find a medium effect size (*d* = 0.50) with 90% statistical power at *p* < 0.05, 86 knees per group for independent samples t-test, 90 for Mann–Whitney U test, and 44 total for one sample mean test were required.

In testing the interrater agreement, we interpreted the intraclass correlation coefficient (ICC) as acceptable (> 0.5), moderate (> 0.6), good (> 0.7), high (> 0.8), and excellent (> 0.9) [37].

## Results

The average age was 34.2 ± 11.2 years (range 18–60) with no difference between males (32.1 ± 11 years) and females (36.2 ± 11.1 years) (*p* = 0.65).

Both offsets and the lateral offset ratio were larger in males (*p* < 0.001), the medial offset ratio, and the medial slope in females (*p* from < 0.001 to 0.007), whereas the lateral slope was sex-free (*p* = 0.41) (Table 1). Irrespective of sex, however, the medial offset with its ratio and the medial slope were larger than their counterparts (*p* < 0.001) (Table 2).

**Table 1:** Posterior condylar offset and the posterior condylar offset ratio of the femur, as well as the posterior slope of the tibia on each side with regard to sex

Sex	Parameters (mean ± SD)						
	*n*	OL	OM	RL	RM	SL	SM
Male	100	24.6 ± 1.9	27.3 ± 2.5	0.4 ± 0.04	0.4 ± 0.04	6.8 ± 2.2	8.0 ± 2.7
Female	100	22.5 ± 2.3	24.3 ± 2.6	0.4 ± 0.04	0.5 ± 0.05	7.0 ± 2.1	9.1 ± 2.8
2-tailed *p*		< 0.001	< 0.001	< 0.001	< 0.001	0.41*	0.007*

*Mann–Whitney–U test, *n*—sample size of a group, OL—lateral posterior condylar offset of the femur (in millimeters), OM—medial posterior condylar offset of the femur (in millimeters), RL—lateral posterior condylar offset ratio of the femur, RM—medial posterior condylar offset ratio of the femur, SL—lateral posterior slope of the tibia (in degrees), SM—medial posterior slope of the tibia (in degrees)

**Table 2:** Posterior condylar offset and the posterior condylar offset ratio of the femur, as well as the posterior slope of the tibia with regard to side

Side	Parameters (mean ± SD)			
	*n*	O	R	S
Medial	200	25.8 ± 2.9	0.5 ± 0.04	8.5 ± 2.9
Lateral	200	23.5 ± 2.4	0.4 ± 0.04	6.9 ± 2.8
2-tailed *p*		< 0.001	< 0.001	< 0.001

*n*—sample size of a group, O—posterior condylar offset of the femur (in millimeters), R—posterior condylar offset ratio of the femur, S—posterior slope of the tibia (in degrees)

The comparison of the offset, the offset ratios, and the slope from various studies is shown in Tables 3 and 4. Where applicable, means of both offsets and their ratios differed between ours and various studies (*p* from < 0.001 to 0.004), slopes included (*p* from ≤ 0.001 to 0.003) except for the medial slope in the study by Zhang et al. [38] (*p* = 0.51).

**Table 3:** Values of the posterior condylar offset and the posterior condylar offset ratio of the femur in the literature

Authors	Population	Method	Sample size	OL	OM	RL	RM	Class
Wang et al. [19]	Chinese	CT	100	27.3 ± 2.3	27.3 ± 2.3	0.5 ± 0.03	0.5 ± 0.03	M
			(50 M, 50 F)	25.8 ± 2.7	25.8 ± 2.7	0.5 ± 0.04	0.5 ± 0.04	F
				26.6 ± 2.5	26.6 ± 2.5	0.5 ± 0.03	0.5 ± 0.03	C
Koh et al. [18]	Korean	MRI	975	24.8 ± 2.4	26.8 ± 2.3	0.4 ± 0.06	0.4 ± 0.07	M
			(150 M, 825 F)	24.2 ± 2.2	26.2 ± 2.2	0.5 ± 0.04	0.5 ± 0.04	F
				24.3 ± 2.3	26.3 ± 2.2	0. 5 ± 0.05	0.5 ± 0.05	C
Voleti et al. [39]	49 Caucasian,	MRI	100	27.0 ± 2.0	30.0 ± 2.5	0.4 ± 0.05	0.5 ± 0.04	M
	27 African American,		(50 M, 50 F)	25.0 ± 2.0	28.0 ± 2.7	0.4 ± 0.03	0.5 ± 0.05	F
	16 Asian, 8 Hispanic			26.0 ± 2.2	29.0 ± 2.8	0.4 ± 0.04	0.5 ± 0.05	C
Weinberg et al. [8]*	366 Caucasian	Cadaveric	529 (461 M,	31.6 ± 3.5	32.9 ± 4.1	1.1 ± 0.2	1.2 ± 0.2	M
	163 African American		61 F) cadaver,	30.2 ± 3.1	32.7 ± 4.4	1.1 ± 0.1	1.7 ± 0.2	F
			1058 femora	31.2 ± 4.1	32.6 ± 3.8	1.1 ± 0.2#	1.2 ± 0.2#	C
Johal et al. [35]	Western (84.0% white,	X-rays	100	29.0 ± 2.0	29.0 ± 2.0	0.4 ± 0.02	0.4 ± 0.02	M
	6.0% black, 5.5% Asian, 3.6% mixed		(50 M, 50 F)	27.2 ± 2.2	27.2 ± 2.2	0. 5 ± 0.02	0.5 ± 0.02	F
	race, 3.0% Arabic 0.3%, 0.6% other)			28.1 ± 2.3	28.1 ± 2.3	0.4 ± 0.02	0.4 ± 0.02	C
Our study	Egyptian	MRI	200	24.6 ± 1.9	27.3 ± 2.5	0.4 ± 0.04	0.4 ± 0.04	M
			(100 M,100 F)	22.5 ± 2.3	24.3 ± 2.6	0.4 ± 0.04	0.5 ± 0.05	F
				23.5 ± 2.4	25.8 ± 2.9	0.4 ± 0.04	0.5 ± 0.04	C

*Values standardized to the patient’s femoral size by dividing actual offset with the anteroposterior diameter of the femur as opposed to that of the femoral condyles hence differing greatly from other studies

#Not applicable for mean comparison, *C* cohort, *CT* computed tomography, *F* female, *M* male, *MRI* magnetic resonance imaging, *OL* lateral posterior condylar offset of the femur (in millimeters), *OM* medial posterior condylar offset of the femur (in millimeters), *RL* lateral posterior condylar offset ratio of the femur, *RM* medial posterior condylar offset ratio of the femur

**Table 4:** Values of the posterior slope of the tibia in the literature

Authors	Population	Method	Sample size	SL	SM	Class
Ho JPY et al. [40]	Asian (38% Indian [South Asia],	CT	100	–	–	M
	25% Chinese [East Asia], and 37%			–	–	F
	Malay [Southeast Asia])			10.9 ± 3.7	11.3 ± 3.2	C
Haddad et al. [41]	Caucasian	MRI	59	–	–	M
				–	–	F
				4.4 ± 4.2	4.2 ± 3.7	C
Haddad et al. [41]	Asian	MRI	37	–	–	M
				–	–	F
				8.1 ± 4.0	7.9 ± 3.7	C
Khattak et al. [42]	Asian	X-rays	59	12.0 ± 3.1	12.5 ± 3.7	M
	(Pakistanis)			11.9 ± 4.5	16.0 ± 3.6	F
				–	–	C
Yue et al. [10]	Asian	CT	40	5.2 ± 3.6	6.0 ± 2.5	M
	(Chinese)			4.8 ± 2.8	5.4 ± 2.3	F
				–	–	C
Zhang et al. [38]	Asian	CT	80	–	–	M
	(South China)			–	–	F
				7.6 ± 2.5	8.4 ± 3.1	C
Our study	Egyptian	MRI	200	6.8 ± 2.2	8.0 ± 2.7	M
				7.0 ± 2.1	9.1 ± 2.8	F
				6.9 ± 2.8	8.6 ± 2.9	C

*C* cohort, *CT* computed tomography, *F* female, *M* male, *MRI* magnetic resonance imaging, *SL* lateral posterior slope of the tibia (in degrees), *SM* medial posterior slope of the tibia (in degrees)

ICCs were high throughout (> 0.8) (Table 5).

**Table 5:** Inter-rater agreement of the MRI measurements

Parameters	OL	OM	RL	RM	SL	SM
Inter-rater agreement ICC (95% CI)*	0.840 (0.785–0.881)	0.813 (0.752–0.825)	0.985 (0.975–0.991)	0.989 (0.983–0.994)	0.889 (0.818–0.942)	0.832 (0.778–0.873)
*p*-Value	< 0.001	< 0.001	< 0.001	< 0.001	< 0.001	< 0.001

*ICC intraclass correlation coefficient, ICC categories (> 0.5 acceptable, > 0.6 moderate, > 0.7 good, > 0.8 high, > 0.9 excellent), *OL* lateral posterior condylar offset of the femur (in millimeters), *OM* medial posterior condylar offset of the femur (in millimeters), *RL* lateral posterior condylar offset ratio of the femur, *RM* medial posterior condylar offset ratio of the femur, *SL* lateral posterior slope of the tibia (in degrees), *SM* medial posterior slope of the tibia (in degrees)

## Discussion

The most important finding of this MRI study was the sexual dimorphism of the posterior condylar offset of the femur and the medial posterior slope of the tibia in non-arthritic knees of Egyptian adults. To the best of our knowledge, no prior studies have measured the two on the Middle Eastern cohort. The current research is, in fact, a continuation of previously published work on the MRI-based anthropometric measurement of the Egyptian non-arthritic knees [16].

Irrespective of sex, the medial offset was larger than its counterpart, a finding supported by multitude of studies [8, 18, 32, 39]. Contemporary TKA designs, however, do not address the issue [21]. Even though some custom implants have managed to do so, a long-term follow-up seems necessary [43, 44]. Addressing this polarity may ensure proper anteroposterior placement of the femoral component which could help avoid notching as well as internal rotation of the femoral component, especially if posterior reference instruments are used.

Sexual dimorphism of the offset was formerly reported by many authors. Koh et al. [18] have shown sex difference of the offset and its ratio with both offsets larger in males and both ratios larger in females which is somewhat in line with male supremacy of our lateral offset ratio. These results, along with other studies disclosing sex differences of the offset and its ratio [7, 19, 32, 34, 45], uphold the necessity of sex-specific femoral component design in reestablishing the offset for males and females in Asian and Middle Eastern populations. Still, various authors [8, 39, 46] presented no sex discrepancy *vis-à-vis* the offset and its ratio. Numerous studies have found no significant difference between sex-specific and standard knee implants regarding clinical and radiological outcomes, patients’ satisfaction and complication rate [31, 47, 48].

Some may argue a sex-specific femoral component might not be essential as the offset may be recreated using a posterior referencing system to perform a measured resection of the posterior condyles of the femur that will preserve any difference between them [18]. Nevertheless, without the utilization of a sex-specific femoral component at a given anteroposterior diameter of the femoral component in use, the offset’s sex-split of only 2–3 mm will either cause overcutting of the posterior offset on one hand or overstuffing with smaller or larger implanted femoral components on the other.

The importance an offset restoration bears on postoperative range of flexion appears controversial and depends on the type of knee replacement. Some researchers advocate the postoperative decrease in the offset by more than 3 mm reduces the postoperative ROM in Cruciate–Retaining (CR) yet not in Posterior–Stabilized (PS) knee implants. It is, therefore, critical to preserve the offset in the CR rather than in PS knees to ensure optimal ROM after surgery [20]. Wang et al. [49] found the offset restoration plays a major role in the optimization of active knee flexion during weight-bearing conditions after PS TKA, with no benefit to non-weight-bearing knee flexion or any other clinical outcome [49]. On the flip side, Ishii et al. [29] found no correlation of individual differences of the offset with current CR or PS prostheses and changes in knee flexion after 1-year follow-up [29]. Chang et al. [27] reported no difference in postoperative offset in Anterior-Referenced (AR) and Posterior-Referenced (PR) group. Moreover, the offset was more consistently preserved after surgery in the PR group. The postoperative offset and ratio changes did not affect the postoperative ROM. Similar clinical outcomes were obtained in the AR as well as PR groups [27].

Weinberg et al. [8] were the first to report Caucasians have greater offsets than African Americans [8]. Wang et al. [19] measured the offset and its ratio in Chinese patients corroborating the former was significantly smaller in both sexes than that of the Western population, as reported by Johal et al. [35]. Conversely, the offset ratio was significantly larger than that of the Western population indicating not only the size of the distal part of the femur differed from the Western population but also its shape. This would imply a Chinese knee having a greater offset at a given anteroposterior diameter of the distal part of the femur. Likewise, Koh et al. observed a smaller offset of the Korean population as opposed to Western population in both sexes, though the former had a larger offset ratio [18, 35]. Therefore, considering the variance of the offsets among different ethnicities, a knee implant design under the axiom of “one-size-fits-all” would inevitably over- or under-restore the offset if used in diverse ethnic groups [8].

On top of that, both slopes were significantly larger in Egyptians as opposed to Caucasians [41] (Table 4). In juxtaposition with Asians, however, the results were variable [42]. The slopes were smaller in the Egyptian population as opposed to Asian population according to Ho et al. [40]. The medial was larger and the lateral slope smaller in Egyptian population than in Asian population as per Haddad et al. [41]. The medial slope was not significantly different, yet lateral was smaller in Egyptian population than its Asian counterpart as reported by Zhang et al. [10, 38].

The restoration of the slope has been shown to delay the tibiofemoral impingement and thus substantially improve the range of knee flexion [50]. It is crucial a prosthetic slope approximates the native as ligaments have been accustomed to the latter [51]. Currently, the optimal slope for the prosthetic knee remains debatable, despite the fact a 0–7 cut is routinely recommended [51, 52]. Following this recommendation, based mostly on the Western populations’ slope, an Egyptian patient may obtain a slope smaller than that prior to arthroplasty, resulting in a tight knee flexion due to magnified tension of the posterior cruciate ligament (if retained), as well as the collaterals [23, 25, 53]. Limited flexion would cause difficulty with regard to activities such as kneeling and squatting, quite frequent in an Egyptian milieu [33, 54].

Our study has certain limitations. The cohort was a single ethnic group which makes the sexual dimorphism of the offset and the slope limited to our geographic area although it may provide a background for comparison with other similar studies elsewhere. We feel more ethnicity-based research is indispensable to begin with. In addition, the measurements were performed on MRIs of non-arthritic knees. This is not a usual stance while performing TKA on arthritic knees. We nonetheless think measurements on healthy knees should act as the foundation in designing new implants for specific populations. Knowing what healthy looks like is, in a nutshell, a mandatory first step to knowledge. Surgeons should, therefore, consider the issue of extra bony cuts in cases of severe deformity during the preoperative planning. Lastly, the absence of a musculoskeletal radiologist among the coauthors might be perceived as a justly limitation. Comparing our results with those formerly reported in other studies might be criticized for being unreliable due to differences in measurement methods, types of imaging, and diverse conditions of the knee joints. Our intent here, however, was not to perform a meta-analysis, but rather to corroborate our review of the current literature with a relatively meaningful statistical calculus. Our study does merit some advantages, of course, such as a large cohort measured by two raters on the MRI as well as favorable statistical power (≥ 90%). The MRI has demonstrated superb capabilities in measuring the offset as it can appraise the thickness of the articular cartilage of merely ≈ 2.75 mm [55], even 2.15 mm [56] according to some reports [57]. Radiographic measurement underestimates the offset due to positioning of the knee and transparency of articular cartilage, hence possibly instilling debate on its importance in TKA [39]. MRI consequently provides a more precise anatomical measurement as the bedrock of prospective knee implant design.

## Conclusion

There was a sexual dimorphism of the posterior condylar offset of the femur and the medial posterior slope of the tibia in non-arthritic knees of Egyptian adults. This would indicate most of the commonly used knee implants, even those sex specific, may not provide a perfect fit unless sex and ethnic variations in both the offset and the slope were previously assessed. We believe future designs of knee implants should consider these differences in order to improve postoperative ROM and patients’ satisfaction after TKA.

## Data Availability

The datasets generated during and/or analyzed during the current study are available from the corresponding author on reasonable request.

## Abbreviations

AR: Anterior referenced
BMI: Body mass index
CR: Cruciate retaining
CT: Computed tomography
ICC: Intraclass correlation coefficient
MRI: Magnetic resonance imaging
PR: Posterior referenced
PS: Posterior stabilized
ROM: Range of motion
TKA: Total knee arthroplasty
